# Benfluorex and Unexplained Valvular Heart Disease: A Case-Control Study

**DOI:** 10.1371/journal.pone.0010128

**Published:** 2010-04-12

**Authors:** Irène Frachon, Yves Etienne, Yannick Jobic, Grégoire Le Gal, Marc Humbert, Christophe Leroyer

**Affiliations:** 1 Université Européenne de Bretagne, Brest, France; 2 Université de Brest, EA3878 (GETBO) IFR 148, Brest, France; 3 CHU de la Cavale Blanche, Groupe HTAP de Bretagne Occidentale Département de médecine interne et de pneumologie, Brest, France; 4 CHU de la Cavale Blanche, Département de cardiologie Groupe HTAP de Bretagne Occidentale, Brest, France; 5 Université Européenne de Bretagne, Brest, France; 6 Université de Brest, INSERM CIC 05-02 IFR148, Brest, France; 7 Université Paris-Sud, Faculté de Médecine, Kremlin-Bicêtre, France; 8 Assistance Publique - Hôpitaux de Paris, Centre National de Référence de l'Hypertension Pulmonaire Sévère Service de Pneumologie et Réanimation Respiratoire, Hôpital Antoine Béclère, Clamart, France; 9 INSERM U999, Hypertension Artérielle Pulmonaire: Physiopathologie et Innovation Thérapeutique, Le Plessis-Robinson, France; York University, Canada

## Abstract

**Background:**

Recent case reports suggest that benfluorex, a fenfluramine derivative used in the management of overweight diabetic patients and dyslipidemia, is associated with cardiac valve regurgitation.

**Methods:**

We conducted a case-control study. Eligible patients were those admitted in the cardiology or the cardiac surgery units of our hospital between January, 1^st^ 2003 and June 30^th^ 2009, with mitral insufficiency diagnostic codes (ICD-10 I340 and I051). Patients with either a primary cause (degenerative, known rheumatic heart disease, infectious endocarditis, congenital, radiation-induced valvular disease, associated connective and/or vasculitis disease, trauma, tumor) or a secondary (functional) cause were considered as having an “explained” mitral regurgitation. Other patients were considered as having an “unexplained” mitral regurgitation and were included as cases. For each case, two controls were matched for gender and for the closest date of birth, among a list of patients with an “explained” mitral regurgitation. Drug exposures were assessed blindly regarding the case or control status, through contacts with patients, their family and/or their physicians.

**Results:**

Out of the 682 eligible patients, 27 cases and 54 matched controls were identified. The use of benfluorex was reported in 22 patients: 19 of the 27 cases, versus 3 of the 54 controls, odds-ratio 17.1 (3.5 to 83), adjusted for body mass index, diabetes and dexfenfluramine use.

**Conclusion:**

The use of benfluorex is associated with unexplained mitral regurgitation.

## Introduction

Exposure to fenfluramine and dexfenfluramine has been associated with the occurrence of cardiovascular side effects, including pulmonary arterial hypertension (PAH) and valvular heart disease [1], [2]. As a consequence, these two anorexigens were withdrawn from drug market in 1997. Meanwhile, involvement of 5-HT_2B_ receptors in fenfluramine-induced valvular heart disease has been demonstrated, and the medical community was warned of the possible fenfluramine-like cardiovascular side effects of potent 5-HT_2B_ receptor agonists [3]. Recently, we reported five PAH cases and one severe valvular heart disease in patients exposed to benfluorex [4], a fenfluramine derivative [5]. This drug has been marketed since 1976 in Asia, Europe and South America and administered for the treatment of overweight diabetic patients and dyslipidemia. It is still the object of active research, including two ongoing clinical trials (WHO International Clinical Trials Registry Platform). As it was currently used by about 300,000 patients each year in France according to the national insurance database, our observations, added to two previous case reports in the literature [6], [7], prompted us to conduct a retrospective case-control study in order to examine the possible association of benfluorex exposure with mitral regurgitation, one of the cardiovascular conditions described in patients exposed to fenfluramine derivatives [2], [8].

## Methods

We designed a retrospective case-control study. Eligible patients were those admitted to either the cardiology or cardiac surgery units of Brest University Hospital between January 1^st^, 2003 and June 30^th^, 2009. Using our institution patient discharge data file, we chose to focus on mitral regurgitation and retrieved patients' charts with “non-rheumatic or rheumatic mitral (valve) insufficiency” (International Classification of Diseases, 10-th Revision, I340 and I051). The study was conducted according to French regulations and approved by our institutional Ethics Committee (Comité d'éthique du CHRU de Brest). As requested by the Ethics committee, a verbal consent was obtained prior to the phone interview.

### Ascertainment of disease

We classified the patients into two groups: with and without an identifiable cause of mitral regurgitation.

The first group had a valvulopathy with a primary or secondary cause recorded explicitly in the patient's hospital file. Primary cause was: either degenerative (mitral-valve prolapse with cordal rupture, annular calcification), known rheumatic heart disease, infectious endocarditis, congenital, radiation-induced valvular disease, associated connective and/or vasculitis disease, trauma or tumor. Secondary (functional) cause was defined by a structurally normal mitral valve on echocardiography and the presence of an underlying cardiomyopathy i.e. either dilated cardiomyopathy responsible for a mitral annulus dilatation, or ischemic left-ventricular remodeling or hypertrophic cardiomyopathy (primary obstructive cardiomyopathy or secondary to severe aortic restriction) responsible for loss of coaptation. The ischemic origin of the cardiac disease had to be documented by a coronary angiography.

The second group included patients without any of these etiologies, who were considered as having an “unexplained” mitral regurgitation.

A research nurse was trained to screen patients' charts: medical history, echocardiography report, operative report in patients who underwent valvular heart surgery, and histological report. If one of the conditions mentioned above was listed, the patients were classified as having an explained mitral regurgitation. Whenever the information was unclear, two physicians (YE, YJ) reviewed the data in order to classify the patients regarding valvular heart disease etiology.

### Selection of cases and controls

All patients classified as having an unexplained mitral regurgitation were included as cases. For each case, a list of potential controls was established from the roster of patients with an explained mitral regurgitation. This list included all patients of the same gender, hospital admission unit, and admission date (within a one year period of the case's admission date). Finally, from this list, we selected as controls the two patients with the closest date of birth to that of the case.

### Ascertainment of drug exposure

The assessment of drug exposure was performed similarly for cases and controls. Independently trained research nurses unaware of the patient's status (case or control) called each patient and his/her family physician to collect information about associated conditions (diabetes, obesity, dyslipidemia) and drug exposure (amphetamine derivatives), using a semi-structured questionnaire. Physicians were asked to consult their patient's files during the telephone call. Information collected included the patient's height, weight (current and highest life-time weight) and associated conditions. Patients and physicians were asked whether or not the patient experienced weight-related issues, and whether or not he/she ever used appetite suppressants, weight-loss drugs or slimming diets. Current diabetes medications were recorded. All amphetamine derivative brand names and international non-proprietary names, including dexfenfluramine and benfluorex, were listed during the interview, and patients were asked whether or not they remembered having used one of these medications. Whenever the patient or his/her physician disclosed any use of benfluorex, information on the exposure period was collected. A standardized case-report form was filled for each patient. In the event of death, information was retrieved by interviewing the patient's relatives and family physician. Patients were considered as exposed to benfluorex if the patient and/or the family physician mentioned benfluorex use. All medical records were also checked, and the mitral regurgitation diagnostic date was retrieved.

### Data analysis

The characteristics of cases and controls were described using mean and standard deviation for continuous variables, and proportion for categorical variables. The proportions of exposed patients among cases and controls were compared using a chi-square test. The odds-ratio of the association between benfluorex and unexplained mitral regurgitation was computed along with its 95% confidence interval (95% CI). A multivariate logistic regression model was performed to cater for possible confounders: diabetes and obesity. SPSS software was used in all data processing.

## Results

Between January 1^st^, 2003 and June 30^th^, 2009, 682 patients were admitted at least once to the cardiology department or to the cardiothoracic surgery unit of Brest University Hospital with non-rheumatic or rheumatic mitral (valve) insufficiency. Mean age was 69.8 years (standard deviation 12.7 years), and 385 (56.5%) were males. A total of 636 patients had an explained mitral regurgitation, 27 were unexplained, and 19 patients (2.8%) were unclassified because the information available for ascertainment was not sufficient.

Thus, the final study sample comprised 81 patients. Twenty-seven cases (i.e. all patients with unexplained mitral regurgitation) and 54 gender-, admission unit-, admission date- and age-matched controls, selected among the 636 patients classified as having explained mitral regurgitation. The etiology of mitral regurgitation for the controls patients is shown in Table 1. Two controls needed to be replaced using a new selection, one because neither the patient nor the family physician could be contacted for exposure ascertainment and the other due to a language issue.

**Table 1:** Etiology of the mitral regurgitation in the 54 control patients.

Etiology of mitral regurgitation	N = 54
**Primary mitral regurgitation**	
Mitral valve prolapse with cordal rupture	19
Annular calcification	4
Known rheumatic heart disease	8
Infectious endocarditis	2
Congenital	3
Connective or vasculitis disease	1
Tumor	1
**Secondary mitral regurgitation**	
Left ventricular dilatation	7
Ischemic left ventricular remodeling	6
Hypertrophic cardiomyopathy	3
Other secondary cause	0

The general characteristics of the cases and controls are displayed in Table 2. Most of the patients were females (72 out of 81, 89%). The cases had statistically a significantly higher body mass index than controls: 30.8 kg/m^2^ (standard deviation 8.1) versus 25.2 kg/m^2^ (SD 7.1), p = 0.002, and were more prone to diabetes: 37.0% versus 7.4%, p = 0.002. At the time of the study, 15 patients (6 cases and 9 controls) had died. Ascertainment of drug exposure could be performed in all patients from at least one source (family physician) and information was available from both, the patient (or proxies) and his/her family physician for 85% of the cases and 85% of the controls. Information was provided by proxies in 11% (3/27) of the cases and 13% (7/54) of the controls. Eight of the 10 proxies were interviewed because the patient was dead. There were three discrepancies in exposure assessment between physicians and patients or proxies: one 84-year old woman case, whose physician notified a long exposure to benfluorex, was not able to confirm this information; two proxies of control patients were not able to confirm the absence of benfluorex use reported by the physicians. The last say pertained to physicians in these three instances.

**Table 2:** General characteristics of cases and controls.

Characteristics, m: mean, SD: standard deviation	Cases (n = 27)	Controls (n = 54)	p-value
Age, years, m (SD)	60.9 (11.5)	63.8 (11.6)	0.29
Female gender, n (%)	24 (88.9)	48 (88.9)	1.00
Body mass index, kg/m^2^, m (SD)	30.8 (8.1)	25.2 (7.1)	0.002
Diabetes, n (%)	10 (37.0)	4 (7.4)	0.002
Mitral valvular surgery, n (%)	14 (51.9)	26 (49.1)	0.81
Deceased, n (%)	6 (22.2)	9 (16.7)	0.54
Interview of both patient (or relative) and GP	23 (85)	46 (85)	1.00
Dexfenfluramine use	9 (33.3)	2 (3.7)	0.001
**Benfluorex use**	**19 (70.4)**	**3 (5.6)**	**<0.001**
	**Odds-ratio** (95% CI): **40.4 (9.7 to 168.3)**		

The use of benfluorex was reported in 22 patients: 19 of the 27 cases (70.4%), versus 3 of the 54 (5.6%) controls, odds-ratio 40.4 (95% confidence interval 9.7 to 168.3), p<0.001. This association remained statistically significant after adjustment regarding body mass index and diabetes: odds-ratios for benfluorex use, body mass index and diabetes were 27.6 (95%Cl 6.1 to 124.6), 1.1 (95%CI: 1.0 to 1.2), and 1.9 (95%CI: 0.3 to 11.7), respectively. There was no interaction between variables included in the logistic regression model.

Overall, 28 patients were identified as users of amphetamine derivatives. Among them, 22 were users of benfluorex, eight of whom also reported a previous use of dexfenfluramine, and one of biphetamine. The six remaining patients (who had never used benfluorex) reported the use of dexfenfluramine alone (n = 3), clobenzorex (n = 1), phenmetrazine (n = 1), and an unspecified amphetamine derivative (n = 1). Further adjustment regarding dexfenfluramine use or any amphetamine derivative use did not alter the results: adjusted odds-ratio for benfluorex use 17.1 (95%CI 3.5 to 83.0) and 21.4 (95%CI 1.6 to 284.9), respectively.

Approximate exposure periods could be obtained in all users of benfluorex. Patients and/or physicians reported the first use of benfluorex before the diagnosis of mitral regurgitation in 18 out of the 19 exposed cases. Among them, 17 were users of benfluorex within the two years before the diagnosis of mitral regurgitation. For all three controls exposed to benfluorex, the reported first use took place after mitral regurgitation was diagnosed. Thus, if we restrict the analysis to exposure occurring before diagnosis of mitral regurgitation, no control patients were exposed (vs 18/27 cases, p<0.0001).

## Discussion

In this case-control study, exposure to benfluorex was reported in 70% of patients with unexplained mitral regurgitation. When compared to a control group of patients with explained mitral regurgitation, a strong association was observed between benfluorex use and unexplained mitral regurgitation: odds-ratio 40.4 (95% confidence interval 9.7 to 168.3). This association was independent from diabetes, body mass index and other amphetamine derivatives use, including dexfenfluramine.

We focused on mitral regurgitation, although other valvular damage, such as aortic regurgitation has been observed with previously investigated fenfluramine derivatives [8]. This choice was based on the fact that almost all the patients in the study by Connolly et al. had mitral regurgitation and mitral regurgitation is a well-characterized condition which is more common than other valvular regurgitations [2], [9]. We selected as cases patients with unexplained mitral regurgitation in order to focus on patients with unrecognized risk factors. We selected as controls patients with an explained mitral regurgitation in order to ensure that cases and controls came from the same population. The selection of controls from patients with “explained” mitral regurgitation has both strengths and weaknesses. Whether selection of controls among patients with the same disease as cases is appropriate or not is a matter of debate. In fact some of the risk factors for “explained” mitral regurgitation may be only weakly associated but any erroneous assumptions in these regards would be expected to dampen any true association. Selecting controls from the general population with diabetes or with metabolic syndrome would have been a far longer process.

We observed a small proportion of unexplained mitral regurgitation, a clinical picture not mentioned in textbooks or in the recent review by Enriquez-Sarano et al [10]. This is consistent with our hypothesis that the possible association between benfluorex exposure and mitral regurgitation should be preferably detectable in the subgroup of patients with no identified risk factor for mitral regurgitation. The classification of mitral regurgitations regarding unexplained and explained etiologies was performed on the basis of routine medical charts. It was impossible to achieve this in only 2.8% of patients. Classification bias is unlikely to have occurred since the whole processing was blinded to drug exposure. Patients were identified through ICD-10-CM codes, irrespective of the severity of mitral regurgitation, and it is difficult to assess this severity retrospectively. However, a mitral regurgitation which is given a diagnosis code in the hospital discharge file is more than likely to be clinically significant. Moreover, a mitral surgical procedure was necessary in half of the cases and the controls.

Benfluorex has been marketed in France since 1976; hence some patients may have forgotten a former use of the medication. However, we asked physicians to check each patient's file for all prior medication use. Because of the retrospective design of the study, precise timing, dose and duration of exposure were not always available, and we could not study the dose-effect relationship. We have focused our main analysis on the use or non-use of benfluorex, without taking into account the timing of the exposure because the data on timing of use and its relation to the exact date of diagnosis was uncertain for some patients. If we limit the exposure to those occurring before the diagnosis of mitral regurgitation, the odds ratio is increased to infinity. Information on drug exposure was collected blindly from the patient's status. A recall bias is unlikely to have occurred since firstly, cases and controls shared the same cardiac condition, and secondly the association between benfluorex and mitral regurgitation was not known. Some patients were also previous users of amphetamine derivatives, mainly dexfenfluramine, and this could have contributed to valve disease. However, dexfenfluramine was withdrawn from the market in 1997, six years before the beginning of our study inclusion period. Therefore, dexfenfluramine is unlikely to have played a role among our patients especially as the short-term effect of dexfenfluramine on regurgitation has been suggested [6]. Moreover, in our study, the association between benfluorex and unexplained mitral regurgitation was independent from dexfenfluramine exposure.

In their landmark study published in 1997, Connolly et al. identifying valvular heart disease in 24 women treated with fenfluramine-phentermine who had no history of cardiac disease, raised the hypothesis of an association between such drug intake and valvular heart disease [2]. One year later, the results of three studies conducted in different settings with different designs were made available: two case-control studies included patients who received fenfluramine derivatives and their matched controls [11], [12]; one trial evaluated the effects of two forms of dexfenfluramine and placebo [13]. All three studies, though differing with regard to the strength of the statistical association and clinical significance, supported an association between the use of fenfluramine derivatives and valvular heart regurgitation. Similarly, following the identification of valvular heart disease in three patients taking pergolide [14], another serotoninergic medication, two case-control studies reported an association between the use of dopamine agonists, pergolide and cabergoline, and valve regurgitation [15], [16]. Meanwhile, evidence for possible involvement of 5-HT_2B_ receptors in the cardiac valvular heart disease associated with ergot derivatives, dopamine agonists and amphetamine derivatives (fenfluramine and methylenedioxymethamphetamine) has been provided [17]. Consequently, practitioners were warned of the possible side effects of potent 5-HT_2B_ receptor agonists [3], [18]. Other drugs belonging to the same class would be expected to carry the same risks, and indeed, with a different patient selection, our study strongly suggests that benfluorex, a fenfluramine derivative marketed in several countries, is associated with valvular heart disease. Arguments in favor of a causal association are the biological mechanism (5-HT_2B_ receptor agonism), the strength and significance of the association, and its independence from other factors.

Based on this study and on the analysis of the situation in France, the French authorities have withdrawn benfluorex from the national market on November 26, 2009 [19].
